# Experimental manipulation of selfish genetic elements links genes to microbial community function

**DOI:** 10.1098/rstb.2019.0681

**Published:** 2020-03-23

**Authors:** Steven D. Quistad, Guilhem Doulcier, Paul B. Rainey

**Affiliations:** 1 Laboratoire de Génétique de l'Evolution, Chemistry, Biology and Innovation (CBI) UMR8231, ESPCI Paris, CNRS, PSL Research University, 10 rue Vauquelin, Paris, France; 2 Department of Microbial Population Biology, Max Planck Institute for Evolutionary Biology, Plön 24306, Germany

**Keywords:** bacteriophages, cellulose degradation, diversity, horizontal gene transfer, ammonification

## Abstract

Microbial communities underpin the Earth's biological and geochemical processes, but their complexity hampers understanding. Motivated by the challenge of diversity and the need to forge ways of capturing dynamical behaviour connecting genes to function, biologically independent experimental communities comprising hundreds of microbial genera were established from garden compost and propagated on nitrogen-limited minimal medium with cellulose (paper) as sole carbon source. After 1 year of bi-weekly transfer, communities retained hundreds of genera. To connect genes to function, we used a simple experimental manipulation that involved the periodic collection of selfish genetic elements (SGEs) from separate communities, followed by pooling and redistribution across communities. The treatment was predicted to promote amplification and dissemination of SGEs and thus horizontal gene transfer. Confirmation came from comparative metagenomics, which showed the substantive movement of ecologically significant genes whose dynamic across space and time could be followed. Enrichment of genes implicated in nitrogen metabolism, and particularly ammonification, prompted biochemical assays that revealed a measurable impact on community function. Our simple experimental strategy offers a conceptually new approach for unravelling dynamical processes affecting microbial community function.

This article is part of the theme issue ‘Conceptual challenges in microbial community ecology’.

## 1. Introduction

Microbial communities underpin all major biological and biogeochemical processes [1]. Appropriate functioning of communities has direct implications for the health of people [2,3], terrestrial [4,5], marine [6] and freshwater [7] ecosystems and even global climate [8]. While the species composition of many communities has been exhaustively documented [9,10], the link between genes and community function is poorly understood [1].

New strategies for investigation are required that are process-focused [11]. Ideally, such approaches will provide knowledge on the complex interconnections between community members, their combined effects, including feedbacks that shape the evolution of community members [12–15]. Recent advances draw upon new strategies for linking patterns of sequence diversity in metagenomic datasets to population genetic processes [16–19]. Knowledge of such processes provides a link between variation and the likelihood that particular traits fix.

Equally desirable are approaches that provide a link between genes, their spatial and temporal dynamics, and community function, achieved using strategies that leave as far as possible the natural complexity of communities undisturbed. Here we show—in a proof-of-principle experiment—that this can be achieved via an experimental strategy that combines theory governing the behaviour of selfish genetic elements [20–23] (SGEs) and expected effects on horizontal gene transfer (HGT), with approaches from experimental evolution [24,25], comparative metagenomics [26] and functional assay.

## 2. Results

### (a) Diversity in experimental communities

Experimental communities were established from a diverse primary source: garden compost. Ten independent 1 g samples of compost were taken from 10 different regions of a single 1 m^3^ compost heap and each placed in one of ten 140 ml bottles (mesocosms) containing 20 ml nitrogen-limited minimal M9 medium plus cellulose (a 4 cm^2^ piece of paper) as sole carbon source. Mesocosms were incubated on a laboratory bench without shaking and with lids left unsealed. After a two-week period, the initial piece of paper from each mesocosm was separately transferred to a new bottle containing fresh M9 medium plus a new piece of paper. Following a further two-week incubation period, we considered the communities to have adjusted to laboratory conditions. At this time point, mesocosms were vortex-mixed and 1 ml of cellulosic slurry from each bottle was transferred to *two* new mesocosms containing 19 ml fresh M9 medium plus paper giving a total of 20 paired communities. This transfer, four weeks after initial establishment, was deemed time point zero (T_0_). Thereafter and for the ensuing 48 weeks, communities were serially propagated every two weeks (T_1_ to T_24_) by transferring 1 ml of slurry to fresh bottles. One set of each pair of bottles was labelled ‘vertical’ (vertical communities (VC)) and the second of each pair labelled ‘horizontal’ (horizontal communities (HC)). Details of the treatments and their significance are elaborated below, but at this stage, with a focus solely on the question of diversity in cellulose-based mesocosms, we acknowledge treatment effects by name only.

**Figure 1.**
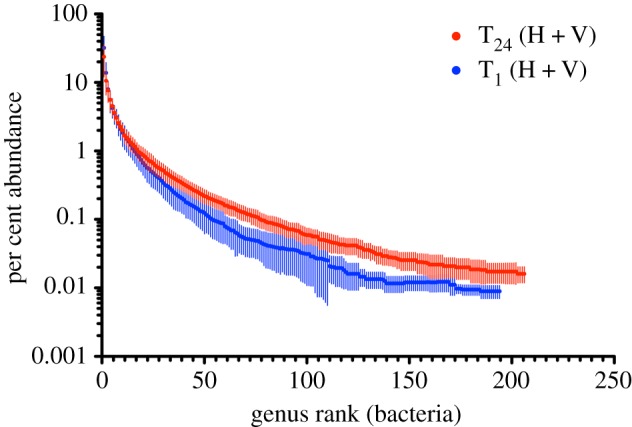
Genus-level rank abundance curves. The blue line depicts the genus-level rank abundance curve for bacteria at time point T_1_ two weeks after the divide of founding communities into horizontal and vertical treatments. The red line is at time point T_24_ (48 weeks). Because there were no differences between VC and HC at T_1_ or T_24_, data from both regimes were combined. Data are means and standard deviations from 20 mesocosms.

Diversity through 48 weeks of propagation was addressed by metagenomics: total DNA was extracted from each of the 10 T_0_, and 20 T_1_ and T_24_ communities and the data interrogated for sequence reads mapping to 16S rDNA (electronic supplementary material, table S1). Communities at T_0_ harboured on average approximately 140 bacterial genera (s.d. ± 23). At T_24_ the number of genera detected increased in all communities to an average of approximately 200 (s.d. ± 32), indicating an increase in abundance of rare types (electronic supplementary material, table S1 and figure S1*a*). Diversity levels were highly similar in both HCs and VCs (electronic supplementary material, figure S1a–c). Rank abundance distributions from pooled HCs and VCs at T_1_ and T_24_ are shown in figure 1 and reveal the tail of the T_24_ distribution to be markedly flatter in both cases, consistent with an overall tendency for rare types to have increased during the course of the year-long selection. Rank abundance distributions for each T_1_ and T_24_ HC and VC, plus the rank order (and change therein) of the most common genera are shown in the electronic supplementary material, figure S2a,b. Electronic supplementary material, figure S3 is a multidimensional scaling plot showing that communities at the start and end of the experiment differed markedly in genera composition and there was no sign of convergence after 48 weeks. Among the most abundant genera, only two harbour species commonly associated with cellulolytic ability [27] (*Cytophaga* and *Cellvibrio*), while others are better known for roles in ammonification [28] (*Azospirillum* and *Paenibacillus* (nitrogen fixation); *Rhodanobacter* and *Pseudomonas* (dissimilatory nitrate reduction/denitrification); electronic supplementary material, figure S2a,b). Single-celled eukaryotes were also present in all mesocosms with several unexpectedly maintaining populations of nematodes through the 48 week selection period.

### (b) Manipulating community-level process

An important goal is the development of strategies that link genes to community function by way of the process. An ideal experiment is one that alters a single ecological or evolutionary process and compares the outcome to a set of replicates in which the process is unaffected. For example, evolutionary biologists interested in the evolutionary consequences of sex have taken advantage of yeast in which it is possible to genetically engineer sexual and asexual types that are otherwise isogenic [29,30]. Differences in evolutionary outcome between sexual and asexual types after a period of selection under identical environmental conditions can thus be attributable to sex.

We reasoned that a similar manipulation might be possible in the context of microbial communities. Recombination mediated by HGT is loosely analogous to sex, albeit to a form of sex that takes place at the level of communities. Moreover, like genetic manipulations affecting sexual reproduction in yeast, the extent of HGT can be controlled by manipulating the evolutionary fate of SGEs [20,23]. Such elements, including phages, plasmids, transposons and integrative and conjugative elements are prime vehicles for the movement of ecologically significant genes among diverse bacteria [31–33]. We note that when SGEs acquire genes that aid host survival they are no longer ‘selfish’ and are more appropriately referred to as ‘mobile’ genetic elements, however, the theory we draw upon comes from thinking about elements that are costly to maintain and thus we continue to use the more generic SGE label.

The critical manipulation is one that affects the likelihood that SGEs encounter new hosts. Frequent exposure to new hosts drives dissemination and evolution of SGEs and linked genes [23]. Conversely, in the absence of new hosts, and thus the opportunity for infectious spread, the selection is powerless to prevent loss of transfer ability leading to degradation of SGEs and thus the reduction in HGT. That loss occurs is evidenced by the remnants of prophages that litter bacterial genomes [34].

**Figure 2.**
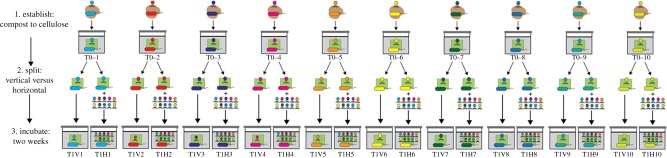
Experimental protocol for manipulation of SGEs and thus the extent of HGT. Cellulose-degrading microbial communities were established by placing 10 independent 1 g samples of fresh compost (taken from separate locations in a single compost heap) into minimal M9 medium contained within a glass mesocosm with paper as the sole carbon source. Following four weeks of incubation at room temperature communities were homogenized and divided in two. Thereafter—and for the next 48 weeks—one of each pair was subject to a ‘vertical’ and the other a ‘horizontal’ transfer regime. The vertical regime involved serial transfer of a sample from each community every two weeks. The horizontal regime involved the same serial transfer protocol, but additionally, at the time of transfer, a sample of supernatant was collected from each of the 10 HCs and passed through a 0.2 µm filter producing an ‘SGE-cocktail’. This sample of 10 SGE-cocktails was pooled (indicated by the 10 different coloured phage cartoons) and redistributed across all horizontal microcosms. Note: DNA was not extracted from the SGE-cocktail prior to mixing (or subsequently).

The *vertical* and *horizontal* treatment designations referred to above reflect manipulations that are predicted to either restrict, or facilitate, respectively, HGT mediated by SGEs. The vertical regime involved serial transfer of material from mesocosm to mesocosm. SGEs within these communities were denied opportunity to encounter new hosts: transfer of SGEs was vertical and the extent of HGT is expected to be limited (figure 2) (and eliminated altogether over longer timescales).

Communities subject to the horizontal regime were transferred by serial dilution to fresh mescosms as per the vertical regime, but received additionally—at the time of serial transfer—an aliquot of ‘SGE-cocktail’. The SGE-cocktail was a pooled sample of SGEs derived from each horizontal mesocosm, which was then redistributed among each HC (figure 2). SGEs within these communities were thus repeatedly (every two weeks) provided with opportunity to encounter new hosts. For example, phages from HC 1 could infect hosts present in HCs 2–9 and may additionally mobilize ecologically significant genes acquired en route. The horizontal regime thus breathes evolutionary life into SGEs and HGT is expected to be rampant.

### (c) Detection of selfish genetic element activity

To determine whether the horizontal regime promoted the movement of SGEs, total DNA was extracted from each T_0_ community and each of the 20 T_1_ communities (two weeks after the split of communities into vertical and horizontal treatments) and sequenced (figure 2 and electronic supplementary material, table S2). Analysis was straightforward given the paired experimental design. For each set of T_0_ and descendant HCs and VCs (e.g. T_0_-1 and descendants T_1_V-1 and T_1_H-1) DNA sequence data were interrogated to identify reads found solely in (and therefore unique to) the horizontal mesocosm (e.g. T_1_H-1). Such unique reads are likely to stem from SGEs, or be associated with SGE activity, originating from an allopatric community.

Analysis of the sequences unique to each horizontal community resulted in identification of approximately 26 million unique reads from a total of approximately 152 million reads across all 10 communities, with an average of approximately 2.6 million unique reads per community (electronic supplementary material, table S4). Assembly of reads into contigs revealed an average of 3352 contigs per HC greater than 1 kb in length. The mean maximum contig size per assembly was 82 kb (electronic supplementary material, table S3).

After extracting open reading frames (ORFs), interrogation of the Conserved Domain Database (CDD) [35] showed 1279 ORFs predicted to encode phage-associated proteins involved in capsid formation, baseplate assembly and phage-induced lysis of bacterial cells (electronic supplementary material, table S3). An example of a contig (T_1_H-1_35969) containing numerous genes characteristic of phages is shown in the electronic supplementary material, figure S4a.

**Figure 3.**
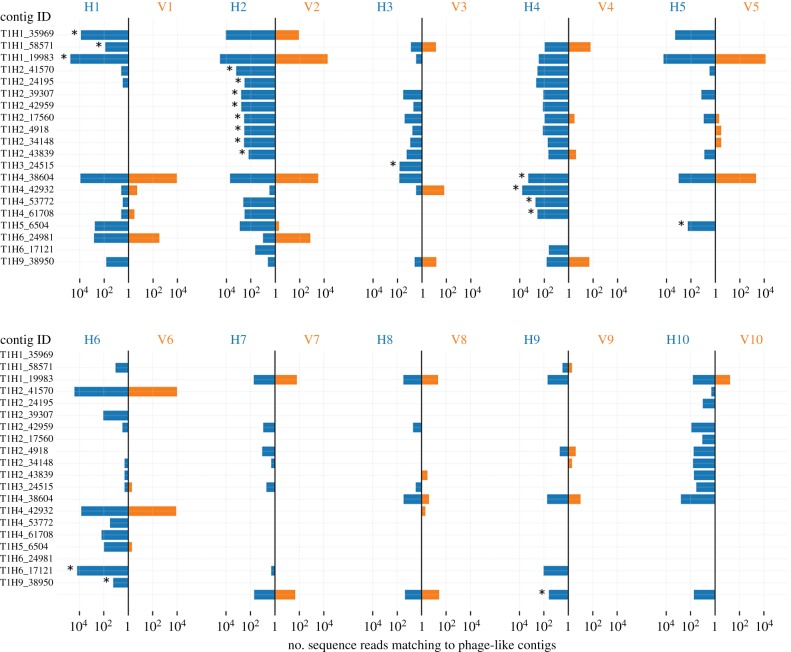
Movement of phage-like elements between HCs. Twenty phage-like contigs assembled from sequence reads unique to HCs are listed on the *y*-axes. Each vertical line represents a single pair of mesocosms with communities subject to the horizontal and vertical regimes depicted in blue and orange, respectively. The *x*-axis of the butterfly plot is the number of reads mapping to each contig. Asterisks denote the source community from which the contigs were assembled.

The distribution and abundance of contig T_1_H-1_35969 and 19 other representative phage-like entities (electronic supplementary material, table S4) across independent mesocosms was determined by mapping total reads from each horizontal and vertical metagenome onto each of the 20 phage-like contigs (figure 3). The predominance of these contigs in HCs is evidence of rapid amplification and dissemination of genetic material. T_1_H-1_35969, originally assembled from the unique set of sequences from T_1_H-1, was also present in both T_1_H-2 and T_1_V-2 and was therefore probably present in the founding community (T_0_-2). Within two weeks, this contig had amplified and spread to HC 1, 5 and 6 (figure 3). Contig T1H2_39307 was below the level of detection in all vertical treatments, but two weeks after imposition of the horizontal treatment, this contig was detected in seven of the independent HCs (figure 3; electronic supplementary material, table S4). From the community perspective, less than four of the 20 phage-like contigs were detected on average per VC, whereas the mean number of contigs per HC was 11 (figure 3).

In addition to phage-like contigs, assemblies of the unique sequences from HCs contained genes predicted to encode traits of likely ecological significance (electronic supplementary material, figure S5). These include genes implicated in cellulose degradation, defence against SGE invasion, nitrogen metabolism and siderophore biosynthesis (electronic supplementary material, table S5) [36–38]. Particularly notable was enrichment of reads mapping to β-glucosidase genes that define the rate-limiting step in cellulose degradation [39]. On occasion, these ecologically significant genes were linked to genes encoding features found on SGEs (electronic supplementary material, table S6), but often these contigs lacked such features (electronic supplementary material, figure S4b). Nonetheless, they were amplified and disseminated through the course of the selection experiment with the same dynamic as expected of a mobile genetic element (see below).

### (d) Tracking the dynamics of selfish genetic element amplification and dissemination

**Figure 4.**
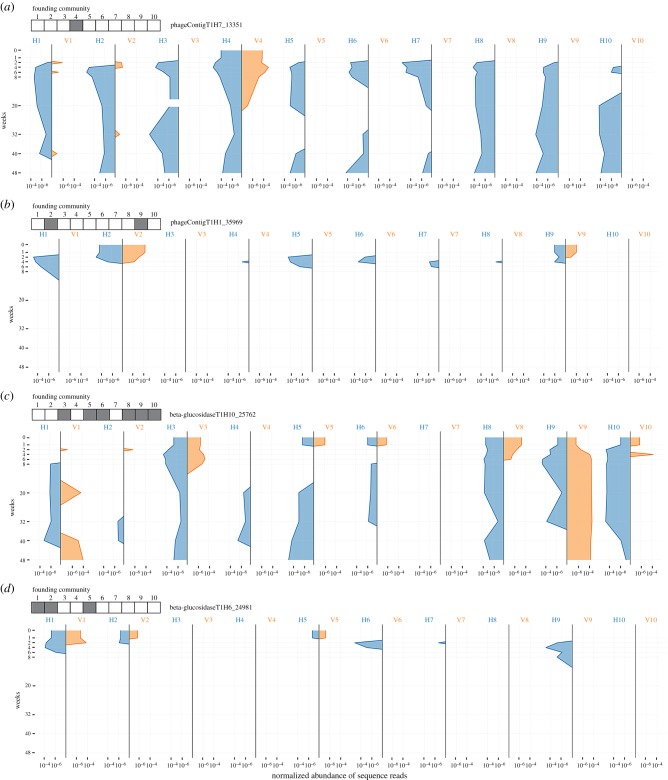
Dynamics of contigs unique to HCs. The dynamics of two-phage-like elements (*a*,*b*) and two elements containing predicted beta glucosidases (*c*,*d*) identified from assembling unique reads from HC at time point T_1_. Each mesocosm is represented as a vertical line and dynamics were tracked through the course of the 48 week experiment. The butterfly plot depicts the normalized abundance of sequence reads mapping to each of the representative contigs from horizontal and vertical mesocosms (blue and orange, respectively). Grey/white boxes above each figure depict presence/absence of sequences from each contig in the founding T_0_ community (before the vertical/horizontal split).

Given evidence indicative of the amplification and dissemination of DNA via the horizontal treatment just two weeks after splitting T_0_ communities into VCs and HCs, we complemented existing DNA sequence from T_0_ and T_1_ and T_24_ with DNA extracted from all HCs and VCs at time points T_2_, T_3_, T_4_, T_10_, T_16_ and T_20_ yielding a total of 180 metagenomes (5.2 billion reads, each approx. 150 bp). These 180 metagenomic datasets (nine time points, 20 communities per time point (electronic supplementary material, table S2)) spread across 48 weeks allowed the abundance and distribution of SGEs and associated genes to be determined by read mapping to contigs assembled from T_0_ data. The spatial and temporal dynamics of two phage-like contigs and two contigs encoding β-glucosidases are shown in figure 4*a–d*.

Focusing on PhageContigT_1_H_7__13351 (figure 4*a*), this phage-like element was detected only in community C4 at T_0_, but was probably present also in communities C1 and C2, although below the limit of detection. By week 3, the phage-like element was present in all HCs. In VCs, the element persisted in community C4 for 22 weeks and transiently in C1 and C2, but was present at 48 weeks in all but one HC. Within HCs, a pattern observed on four occasions saw the phage-like element go extinct (or fall below the level of detection), but re-emerge and persist at latter time points.

### (e) Ecologically significant genes

**Figure 5.**
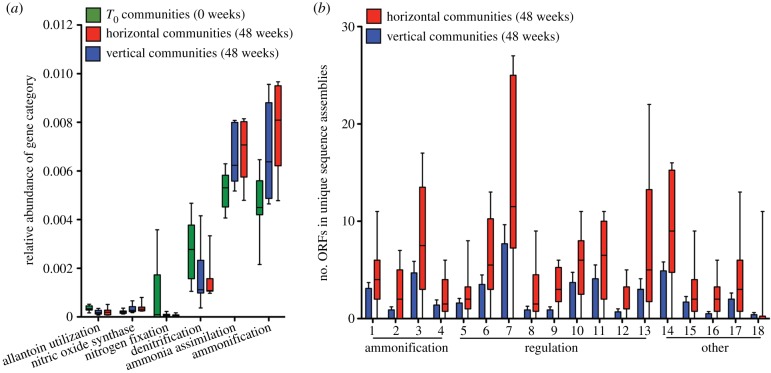
Enrichment of nitrogen metabolism genes in HCs. (*a*) Relative abundance of nitrogen metabolism gene categories based on sequence annotation using MG-RAST. Data are shown as box and whisker plots depicting median, interquartile range (box) and full data spread from 10 replicate communities. (*b*) Total number of ORFs predicted to encode-specific domains associated with ammonification, regulation of nitrogen metabolism and other associated nitrogen metabolism genes in the assemblies of unique vertical and unique horizontal sequences. Data are shown as box and whisker plots depicting median, interquartile range (box) and full data spread from 10 replicate mesocosms. 1, NifS; 2, NifE; 3, NapA; 4, nitrilase; 5, NtrA; 6, NifR3; 7, NtrY; 8, NifL; 9, PtsN; 10, NtrB; 11, NtrC; 12, NAC; 13, FixI; 14, FixG; 15 and 16 nitroreductases; 17, arginase; 18, cytochrome D1. Full descriptions of domains are provided in the electronic supplementary material, table S9.

To determine whether the horizontal transfer regime affected the abundance and distribution of functional genes, the MG-RAST database was interrogated with sequence reads from all T_0_ and all T_24_ HCs and VCs (electronic supplementary material, table S7). For the ensuing analysis, we have avoided standard statistical testing because horizontal microcosms, by virtue of the movement of SGEs and linked genes among communities, cannot be treated as independent entities. Nonetheless, trends are clearly evident from the data displayed in figure 5 (and corresponding electronic supplementary material, figure S6a,b), which include for each functional category—for each vertical, horizontal and T_0_ community—median value, interquartile range and full data range.

The relative abundance of reads assigned to 13 of 28 functional categories at subsystems level 1 [40], including genes involved in virulence, motility and nitrogen metabolism, changed during the course of the year (electronic supplementary material, figure S6a). HCs and VCs did not always change to the same extent, or in the same direction (electronic supplementary material, figure S6a,b). No obvious change occurred in the overall category of carbohydrate metabolism (electronic supplementary material, figure S6b), but an increase in β-glucosidase metabolism—the rate-limiting step in cellulose degradation—was detected in both HCs and VCs (electronic supplementary material, figure S6a).

The nitrogen-limited nature of the culture medium, combined with evidence of selection favouring genes involved in nitrogen metabolism, led to closer focus on this essential resource. The MG-RAST database was again interrogated, but this time at subsystems level 2 [40], which provides information on functional categories within the broader category of nitrogen metabolism (figure 5*a*). Changes were observed in the relative abundance of reads mapping to genes involved in both ammonia assimilation and dissimilation (ammonification). Such changes are consistent with the nature of the selective environment. This prompted a query of the extent to which these changes may have been influenced by the movement of genes via SGEs.

A modified version of the bioinformatic pipeline developed to identify sequences unique to HCs (Methods) was applied to the T_24_ metagenomes. T_24_ HC metagenomes were compared to their paired T_24_ VC metagenomes as well as to all metagenomes from VCs from earlier time points (electronic supplementary material, figure S7a and table S8, Methods figure S1). This showed a greater number of unique sequences in the HCs, but also showed a fraction of unique sequences present in VCs. The latter is to be expected given that T_0_ communities must contain rare sequences that through the course of the selection experiment became common—and is consistent with data on changes in abundance of genera (figure 1). To see whether it was possible to reduce this signal, genomic DNA samples from T_0_ were sequenced on the HiSeq platform resulting in additional 320 million reads per community. The effect of increasing depth of sequence made minimal impact on the detection of unique sequences (electronic supplementary material, figure S7b). Nonetheless, the ‘deep’ T_0_ metagenomes were used in all subsequent analyses.

To determine the fraction of unique reads that mapped to genes involved in nitrogen metabolism, the final sets of unique reads were obtained from HCs and VCs; they were assembled, ORFs identified and functionally categorized using the CDD. HCs contained more unique ORFs predicted to be involved in nitrogen metabolism compared to VCs (electronic supplementary material, figure S8). HCs were enriched in several functional classes of gene, especially those with predicted roles in regulation and ammonification (figure 5*b*; electronic supplementary material, table S9).

**Figure 6.**
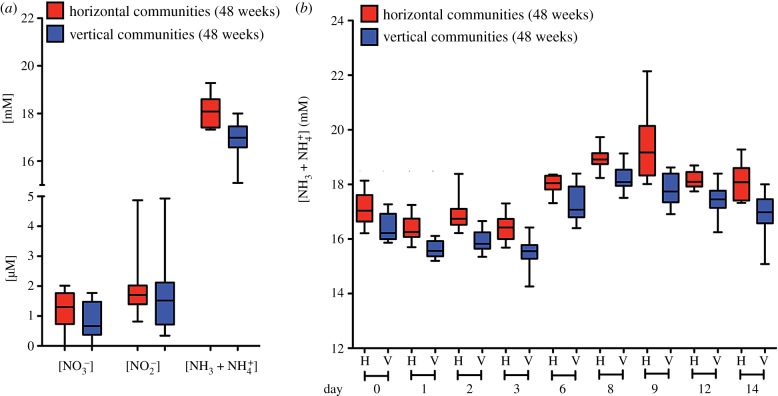
Enhanced ammonia production in HCs. (*a*) Concentrations of nitrate, nitrite, and ammonia/ammonium from mesocosms subject to horizontal and vertical regimes at 48 weeks (T_24_). Data are shown as box and whisker plots depicting median, interquartile range (box) and full data spread from 10 replicate communities. To test for differences in nitrate, nitrite and ammonia/ammonium concentrations at time T_24_ the 10 communities in vertical and horizontal regimes (three independent measures per community) were compared using a two-sample Kolmogorov–Smirnov test (implemented in python 3.7.2, package scipy 1.3.1). Nitrate and nitrite concentration distributions are not significantly different in the horizontal compared to vertical regime (both *p >* 0.1). However, the distribution of ammonia/ammonium concentrations were significantly different (K-S statistic = 0.6, *n* = 30 per sample, *p* < 0.001). Analysis was repeated using a linear mixed effect model. Community identity (1–10) was assigned as a random effect and regime (horizontal/vertical) as a fixed effect (implemented in python 3.7.2, package stats model 0.10.2, restricted maximum-likelihood fit). Results are in agreement with the K-S test. Nitrate and nitrite concentrations were not significantly affected by regime (for both Wald-test results, *p* > 0.3). However, the concentration of ammonia/ammonium was significantly higher in the horizontal regime (mean effect = 1.207, s.d. = 0.151, 95% confidence interval: (0.912, 1.503), *n* = 60, Wald-test, *p* < 0.001). (*b*) Ammonia/ammonium concentrations measured at nine time points during the two-week incubation period for communities from horizontal and vertical regimes at 48 weeks. Data are shown as box and whisker plots depicting median, inter quartile range (box) and full data spread from 10 replicate communities (triplicate measures were obtained from each community on each occasion). Analysis of time-series data using a mixed ANOVA design showed a significant effect of treatment (horizontal versus vertical; *p* = 1.96 × 10^−5^) and a significant effect of time (*p =* 2.7 × 10^−24^, after Greenhouse & Geisser correction for sphericity). The interaction between treatment regime and time was not significant *p* = 0.69). Comparison between the vertical regime at 48 weeks (T_24_) and community function at the beginning of the experiment (T_0_) showed no difference (see the electronic supplementary material, figure S9).

To link to function, we asked whether there was evidence of an effect of the horizontal treatment on a measurable community property. To this end, and with focus on the process of ammonification (production of ammonia through either fixation, or reduction of nitrate/nitrite) the concentration of nitrate, nitrite and ammonia were determined in the T_24_ HCs and VCs at the end of the two-week period immediately prior to serial passage (figure 6; electronic supplementary material, table S10). Bearing in mind the non-independence of HCs, two different, but appropriate statistical tests were employed. The first, a two-sample Kolmogorov–Smirnov (K-S) test revealed no significant difference among HCs and VCs for nitrate or nitrite production, but a highly significant difference in the amount of ammonia produced. The second, a linear mixed effect model, with ‘community’ assigned as a random effect and regime (vertical/horizontal) as a fixed effect, produced results that mirrored those of the K-S test. Details of the tests are provided in the caption to figure 6.

To check the robustness of this finding, the concentration of ammonia was measured during the course of a two-week period. Despite substantive variability in the composition of the independent communities (electronic supplementary material, figures S2a,b and S3), a significantly greater concentration of ammonia was detected in HCs at each of nine sampling occasions throughout the two-week period (figure 6*b*; electronic supplementary material, table S11). A comparison between T_0_ communities and T_24_ VCs showed no change (electronic supplementary material, figure S9) supporting the conclusion that movement of ecologically significant genes via SGEs was responsible for the functional difference between HC and VC.

## 3. Discussion

Simplification is a powerful scientific approach. For example, detailed insight into the dynamics of adaptive evolution has come from the field of experimental evolution, where single (often asexual) genotypes are propagated under defined laboratory conditions with variation arising solely by de novo mutation and where selection is the primary arbiter of mutation success [24,25]. This minimal experimental design avoids mixing (migration) between populations, sex and recombination, thus allowing observed evolutionary change to be attributed directly to specific mutations that can be connected causally to changes in fitness.

We sought to apply the same kind of simplifying experimental approach to the study of microbial communities, however, doing so is not straightforward. Simply substituting a community of microbes in place of a single bacterial genotype, followed by recapitulation of a standard serial transfer experiment, would not be analogous to propagating a single bacterial genotype as done, for example, in the work of Lenski [41]. In the latter, focal populations adapt genetically by natural selection. Substitution of communities for single populations does not result in communities adapting genetically by natural selection: individual bacterial lineages within the community will evolve—although the extent to which their evolution is adaptive is an open and fascinating question [42–44]—but it makes no sense to think about community-level adaptation, because communities (in a standard serial transfer experiment) are not units of selection. To observe adaptation at the community level, communities must participate in the process of evolution by natural selection. This requires the establishment of an experimental regime in which communities are treated as evolving lineages subject to a birth–death process [25,45–48].

If it makes no sense to assume that communities adapt in a manner analogous to single bacterial populations, then attention shifts away from the focus on mutation and selection affecting individual units, and turns to possibilities that might stem from manipulation of evolutionary processes such as recombination, or migration that could conceivably have community-level effects. We chose to manipulate the former, seeing the possibility to promote, or retard HGT, via a simple mixing regime predicted to affect the evolutionary fate of SGEs, which in turn, might connect genes to community function.

Our focal communities were established from a highly diverse environment (garden compost) and propagated on cellulose as sole carbon source. The decision to use cellulose was in part a nod to simplification, but was also motivated by the fact that garden compost is rich in this material, thus ensuring an *in vitro* environment not too dissimilar from that encountered in nature. Additionally, component species can in principle be isolated and cultured on laboratory agar with glucose as sole carbon source (glucose being the utilizable metabolic break-down product of cellulose). However, the primary consideration for use of cellulose was that it is difficult to degrade [27], thus allowing the possibility that communities might be cultured over prolonged periods under nutrient limitation without dominance by *r*-selected types. In effect, while the means and mode of propagation involved serial transfer, communities probably experienced culture conditions more reminiscent of those provided in chemostat culture.

Levels of diversity recorded from analysis of metagenomic data were remarkably high, remaining stable—and even increasing—through the course of the 48 week period of propagation (figure 1). This is unexpected given propagation on a single carbon source, where few types should be stably maintained [49–51]. Were the community propagated on glucose instead of cellulose, almost certainly diversity would have been minimal and dominated by a small number of fast-growing types. That diversity increased over time (figure 1) indicates that rare genera which were initially below the level of detection at the start of the experiment, increased in frequency. The ecological reasons are not known, but point to unanticipated ecological complexity and presumably abundant opportunity for cross feeding.

Although noted only in passing, and from examination of the mapping of DNA sequence reads to genes connected with nutrient cycling (data not shown), cycles for replenishing all major nutrients appeared to have been established within each mesocosm, with that involving nitrogen being most evident. Each community harboured a single dominant free-living nitrogen-fixing bacterium, but the identity—even at the genus level—differed between mesocosms. Attempts to establish such complex communities by choosing key players before the fact, would probably fail. That such diversity and stability can arise from a single gram of compost speaks to an abundance of redundancy in these natural systems [52,53].

Given the experimental design with vertical and horizontal treatment regimes, it is natural to query the relationship between treatment effects and patterns of diversity. At the level of number of bacterial genera and shape of rank abundance curves, no difference was detected and in fact communities showed remarkably similar overall trends. However, differences are apparent when focusing on the rank of individual genera. These are evident in the butterfly plots in the electronic supplementary material, figure S2b and show difference both between independent communities but also differences between vertical and horizontal treatments within communities. Because our experimental design did not include technical replicates (a decision made based on the need to balance experimental feasibility with intuition that we should maximize the number of independent communities) no statistical analysis is possible. Nonetheless, notable is the similarity of changes in genus rankings between vertical and horizontal treatments from the same communities. Also notable is the fact that there was no evidence of convergence in diversity in the horizontal treatment after 48 weeks (electronic supplementary material, figure S3). Although unsurprising, this emphasizes the distinctness of the communities despite being connected via movement of SGEs and linked genes.

The pooling of material from each mesocosm that passed through a 0.2 µm filter to produce an SGE-cocktail, followed by its mixing and regular distribution among HCs, was evidently effective in mobilizing SGEs and promoting horizontal transfer of genes of ecological significance. In fact, the magnitude of the effect, combined with capacity to detect it via identification of DNA sequence reads unique to each horizontal community, exceeded expectation. Within just two weeks of implementation, approximately 17% of DNA sequence reads within HCs were derived from allopatric communities. That these reads could be assembled into contigs containing genes of ecological relevance, including those involved in cellulose degradation, emphasizes the dynamism of processes driven by SGEs.

One consequence of this dynamism is the application of the protocol to detect—at the community level—traits under selection. The suite of unique DNA sequences amplified and disseminated across communities suggests that these genes matter. In the context of conditions experienced by the compost-derived communities, this makes sense. Community members are expected to have experienced selection for the ability to degrade cellulose: this is indicated by the abundance of horizontally transferred genes predicted to be involved cellulose degradation (electronic supplementary material, figure S5). Similarly, as mesocosms were nitrogen-limited, selection was expected to favour the movement of genes involved in nitrogen metabolism. That this happened is indicated by the prevalence of genes implicated in nitrogen metabolism among the set of DNA sequence reads unique to HCs. Not appreciated at the outset was that communities, according to the signature acquired from unique sequence reads, were iron limited. With hindsight, this was to be expected. That genes encoding CRISPR are also enriched is especially significant and suggests an unappreciated dynamic between hosts, the defence systems maintained to protect bacterial hosts against phages, and benefits that accrue from the horizontal dissemination of these systems [54].

Without further experimentation, the identity of the elements responsible for horizontal spread of genes is unknown, and so too is the relationship between the genes amplified and disseminated across mesocosms, and the vehicles for their dissemination. While some assembled contigs show features typical of phages, many do not. This may be a consequence of working with metagenomic data, but may also reflect the existence of elements whose nature and dynamic remains to be discovered.

One of the larger assembled contigs shown in the electronic supplementary material, figure S4b is a case in point: at approximately 80 kb, hints as to its identity are expected, and yet while carrying genes predicted to encode glycoside hydrolyses—implicated in cellulose degradation—the only suggestion of ability to be both mobilized and disseminated is via linkage to a single integrase. Acquisition of long-read sequence data may aid understanding, but it is also possible that the captured dynamic—the rapid amplification and dissemination of ecologically significant genes—is attributable to ‘fortunate accidents’ mediated by, for example, lytic phages or other elements that happen on rare occasion to capture genes of utility to hosts, with the feedback between genes that prove useful to hosts and the amplifying effects of selection driving their dynamic. This points to the possibility of processes that unfold at the level of diverse communities that remain to be understood.

Irrespective of the nature of the disseminating vehicles, the experimental approach outlined allows the dynamics of elements amplified and disseminated to be tracked. Examples are shown in figures 3 and 4. Although mechanistic explanations are unavailable, the dynamics of PhageContigT_1_H7_13351 (figure 3*a*) raise numerous questions. Extinction within VC4, but persistence in all HCs at 48 weeks (with the exception of H1) raises questions as to the causes. Using recently developed Hi-C [16,19,55] and population genetic approaches [18], it ought to be possible, in the future, to disentangle ecological from evolutionary effects. Nonetheless, that persistence is evident in HCs, points to a significant role for SGEs.

The association established between genes involved in nitrogen metabolism and enhanced activity of SGEs in HCs is correlative, but is nonetheless linked to horizontally transferred sequences that map to genes with predicted roles in ammonification. The association is further linked to data that demonstrate a significant effect of the experimental treatment on concentrations of ammonia in HCs (figure 6), but no effect (compared to T_0_ communities) in VCs (electronic supplementary material, figure S9). Additionally, increased ammonia in the HCs is consistent with the prediction that the horizontal movement of DNA will increase the rate at which community function improves.

The two processes that generate ammonia: nitrogen fixation and nitrate ammonification, both require environments devoid of oxygen (or mostly so in the case of nitrate ammonification) [28,56]. It is possible that enhanced metabolic activity associated with cellulose degradation causes lower oxygen conditions at the paper surface (where community members grow as biofilms) compared to the VCs, leading to enhanced production of ammonia. Intriguingly, the cattle industry promotes the addition of ammonia to feed because of beneficial effects on digestibility of plant matter in the rumen [57]. This raises an alternate possibility, which is that elevated ammonia levels are similarly beneficial to the digestion of cellulose in the experimental mesocosms, and reflect a more rapid response to selection in the HCs.

The genomic era has done much to resolve the controversy surrounding the importance of HGT as a driver of evolutionary change [58–60]. Direct evidence of HGT—many facilitated by SGEs—from one organism to another are common [61–64], and the possibility that the process assumes far greater evolutionary significance when operating within communities is currently a topic of discussion [65]. That the vast diversity of DNA sequence encompassed within complex microbial communities might exist in fluid association with SGEs generating permutations of effects significantly beyond those observed through the study of individual SGEs and their hosts, is both interesting and plausible.

Microbial communities are dynamical systems defined by complex ecological interactions and evolutionary feedbacks [1]. The challenges associated with understanding this complexity are immense. Here we have demonstrated the use of an approach in which we have employed a simple experimental manipulation predicted (and shown) to fuel the evolutionary life of SGEs and thus the process of HGT. In effect, mixing (or not) SGEs among communities promotes (or limits) what might be considered community-level sex. The manipulation affects the abundance and distribution of genes of ecological significance, whose individual dynamics can be followed. Further, the effects are sufficient to connect—at a correlative level—genes to community function. That this involves nothing more than frequent exposure of SGEs to new hosts, combined with metagenomic analysis, means that the experimental strategy can be readily transferred to a wide range of microbial communities, from experimental communities as here, through microbiomes of plants and animals, to naturally occurring marine and terrestrial communities.

## 4. Methods

### (a) Establishment of cellulose-degrading microbial communities

Microbial communities were initially established by placing 10 independent 1 g samples of fresh compost into 20 ml of M9 minimal media (the nitrogen source was ammonium chloride (0.935 mM)) supplemented with a 4 cm^2^ piece of cellulose paper (Whatman cellulose filter paper). All incubations were performed in 140 ml sterile glass bottles with loosened screw caps allowing for gas exchange between the community and the environment. Incubation was at room temperature without shaking. Compost was sampled from the Square Theodore-Monod compost heap (Paris, France) in February 2016. During the primary incubation period of two weeks, the piece of cellulose was suspended by a wire from the screw cap into the media allowing for paper colonization. After two weeks, the community was transferred by moving the wire-suspended cellulose paper into 20 ml of fresh M9 minimal media that also contained a new 4 cm^2^ piece of cellulose paper in suspension. Two more weeks of incubation provided the opportunity for colonization of the new piece of suspended cellulose paper thus eliminating the need for a wire-suspended community for each transfer. All community transfers were performed by vortexing each bottle at maximum speed until the cellulose fibres were disaggregated into a slurry.

### (b) Horizontal and vertical transfer regimes

Vertical and horizontal transfers were performed at exactly the same time every two weeks. Before each transfer aliquots of the community were taken for glycerol stocks, DNA extraction and phage storage. Glycerol stocks were created by mixing 500 µl of cellulose-microbial slurry with 500 µl of 80% glycerol and stored at −80°C. For DNA extractions 2 ml of cellulose-microbial slurry was centrifuged at 13 000*g* for 10 min and the pellet was stored at −80°C for later processing. DNA was extracted from the founding T_0_ communities (before the horizontal/vertical split) as well as transfers 1, 2, 3, 4, 10, 16, 20, 24 (T_1_, T_2_, T_3_, T_4_, T_10_, T_16_, T_20_, T_24_) using the soil DNA extraction kit (Norgen Biotek). DNA sequencing was performed using the MiSeq and NextSeq platforms for all time points and T_0_ communities with additional deep sequencing of the T_0_ communities using the HiSeq platform.

Transfers at two-week intervals were performed as described in figure 2. The ‘SGE-cocktail’ was prepared by mixing 1 ml of homogenized community (*n* = 10) followed by centrifugation and passage through a 0.2 µm filter to remove microbial cells. Recognizing the possibility that ultra micro-bacteria might escape sedimentation and pass through the filter, metagenomic sequence reads were recruited to seven available reference genomes. A maximum of 0.018% of reads across all horizontal and vertical mesocosms at 48 weeks matched these reference genomes (electronic supplementary material, figure S10).

### (c) Bioinformatic analysis

DNA sequences were demultiplexed using bcl2fastq, paired ends were joined using FLASh [66] and initial fastq files were generated. Preprocessing of fastq files was performed using PrinSeq [67] with a minimum length of 100 base pairs, minimum quality score of 25, and a maximum percentage of N's of 10%. All metagenomes were uploaded to the MG-RAST metagenomic analysis server and are publicly available (electronic supplementary material, table S2). Gene category abundances (electronic supplementary material, figure S5) and genus identifications (electronic supplementary material, table S1) were determined using the MG-RAST pipeline [68]. Sequence matches were determined using BLASTn [69] with a minimum e-value threshold of 1 × 10^−5^, minimum alignment length of 100 base pairs, and a minimum percentage identity of 90%. ‘Unique’ horizontal and vertical sequences were assembled using MEGAHIT [70], ORFs with a minimum length of 100 amino acids were extracted using getORF [71], compared to the CDD [35] using RPS-BLAST, and top domain hits were extracted (electronic supplementary material, table S5). Phage-like contigs were classified based on phage-specific protein domains (electronic supplementary material, table S4) and their distribution was determined using Fr-Hit [72]. Sequences matches to phage-like contig were based on a minimum alignment length of 100 base pairs with a percentage identity of at least 90%. Nitrogen-related genes were based on gene function classifications and descriptions with a total of 136 domains (electronic supplementary material, table S9). To identify unique sequences, a custom bioinformatic pipeline was developed (electronic supplementary material, Methods figure S1). Each horizontal metagenome was compared to the founding T_0_ metagenome (before the horizontal versus vertical split) and the paired vertical metagenome using BLASTn. Horizontal-T_0_ and horizontal–vertical sequence matches were removed, thus leaving a set of unique horizontal sequences that could not be identified in neither the founding T_0_-metagenome, nor the paired vertical metagenome. Sequence matches were determined using a minimum e-value threshold of 1 × 10^−5^, minimum alignment length of 100 base pairs, and a minimum per cent identity of 90%. The unique horizontal sequences were then assembled, ORFs were extracted, and predicted protein domains were determined through comparison to the CDD. To determine the impact of increasing the sequencing depth of the T_0_ communities on the final percentage of unique sequences, T_0_ metagenomes were sequenced using the HiSeq platform and reads from each mesocosm split arbitrarily into eight metagenomes each consisting of about approximately 40 million sequences. The unique analysis pipeline was then applied using the first arbitrary T_0_ metagenome and any matching sequences were removed. The process was repeated with all eight T_0_ arbitrarily defined metagenomes thus revealing the extent to which additional sequencing affected the ability to detect rare sequences.

### (d) Nitrogen assays

Vertical and HC from the 1-year time point (24 transfers, T_24_) and T_0_ were re-established by placing 100 µl of glycerol stock into 20 ml of M9 minimal media supplemented with a 4 cm^2^ piece of cellulose paper and incubated for two weeks. One millilitre of the cellulose-community slurry was transferred to 19 ml of M9 minimal media with a fresh piece of cellulose and incubated for an additional two weeks. One millilitre of the community was then transferred to a new bottle and 100 µl of surrounding media was sampled at various time points during the two-week incubation period to determine ammonia/ammonium, nitrate and nitrite concentrations. The concentrations of all three nitrogen species were determined using fluorometric assay kits according to the manufacturer's protocol (Ammonia Assay Kit, Sigma-Aldrich, and nitrate/nitrite fluorometric assay kit; figure 6 and electronic supplementary material, tables S10 and S11).

## Supplementary Material

Supplementary figures

## Supplementary Material

Supplementary tables

## Data accessibility

All metagenomes are publicly available here: https://www.mg-rast.org/mgmain.html?mgpage=project&project=mgp18485. Electronic supplementary material, table S1 contains metagenome descriptions and MG-RAST identifiers. All associated datasets are publicly available here: https://zenodo.org/record/3293312 and electronic supplementary material, table S12 contains file descriptions and associated metadata
